# Parental touch reduces social vigilance in children

**DOI:** 10.1016/j.dcn.2018.05.002

**Published:** 2018-05-09

**Authors:** Eddie Brummelman, David Terburg, Miranda Smit, Susan M. Bögels, Peter A. Bos

**Affiliations:** a Research Institute of Child Development and Education, University of Amsterdam, University of Amsterdam, P.O. Box 15780, 1001 NG Amsterdam, the Netherlands; b Department of Psychology, Stanford University, United States; c Department of Experimental Psychology, Utrecht University, the Netherlands

**Keywords:** Parental touch, Social vigilance, Social anxiety

## Abstract

The sense of touch develops in utero and enables parent-child communication from the earliest moments of life. Research shows that parental touch (e.g., licking and grooming in rats, skin-to-skin care in humans) has organizing effects on the offspring’s stress system. Little is known, however, about the psychological effects of parental touch. Building on findings from ethology and psychology, we propose that parental touch—even as subtle as a touch on the shoulder—tells children that their environment is safe for exploration, thus reducing their social vigilance. We tested this hypothesis in late childhood (ages 8–10) and early adolescence (ages 11–14) in 138 parent-child dyads. Parents were randomly assigned to touch or not touch their child briefly and gently on the shoulder, right below the deltoid. Parental touch lowered children’s implicit attention to social threat. While parental touch lowered trust among socially non-anxious children, it raised trust among those who needed it the most: socially anxious children. The effects were observed only in late childhood, suggesting that parental touch loses its safety-signaling meaning upon the transition to adolescence. Our findings underscore the power of parental touch in childhood, especially for children who suffer from social anxiety.

## Highlights

• The sense of touch enables parent-child communication from birth onward.
• We theorized that parental touch would reduce children’s social vigilance.
• Parental touch reduced children’s attention to social threat.
• Parental touch increased trust, specifically among socially anxious children.
• These effects occurred before (not after) the transition to adolescence.

## 1 Introduction

The sense of touch relies on human’s largest sense organ, the skin, and is the first of all senses to develop in utero (Field, 2014; Tobin, 2011). From the very moment children are born, parents communicate with them through touch. Unsurprisingly, then, the skin is considered a social organ (Dunbar, 2010; Morrison et al., 2010). Parental touch, such as licking and grooming in rats and skin-to-skin care in humans, has organizing effects on the offspring’s stress system (Feldman, 2011; Field, 2014; Hertenstein et al., 2006; Liu et al., 1997; Weaver et al., 2004). To date, little is known about the psychological effects of parental touch. We propose that parental touch—even as subtle as a touch on the shoulder—communicates to children that their environment is safe for exploration, thus reducing their social vigilance.

Our theorizing builds on ethological and psychological work. Harlow (1958) discovered that when infant macaque monkeys were brought into an unfamiliar environment, they used a surrogate mother made of cloth—rather than one made of wire—as a source of security. Bowlby (1969/1982)Bowlby (1969/1982) noted that even a “light touch” by parents can make children feel safe (p. 261), and thus encourage them to explore the environment. Experimental research in humans concurs with these observations (Jakubiak and Feeney, 2016a). When college students were touched on the shoulder by an experimenter, they reported more feelings of security and were more willing to take risks (Levav and Argo, 2010; also see Jakubiak and Feeney, 2016b; Koole et al., 2013). When married women held hands with their husbands, they showed a reduced neural response to threat, especially if their marriage was of high quality (Coan et al., 2006). However, these studies did not involve parents and children. In one study that did (Feldman et al., 2010; also see Stack and Muir, 1992), mothers interacted with their infant child, and then suddenly maintained a still face—a stressful experience for infants. While maintaining this still face, some mothers were instructed to touch their child “in whichever way they chose” (p. 273). When they did, their child fussed less, cried less, and displayed lower physiological reactivity: attenuated cortisol reactivity, quicker cortisol recovery, and lower cardiac vagal tone.

These studies provide preliminary support for the idea that parental touch signals safety. We hypothesized that, by signaling safety, parental touch would reduce children’s social vigilance: their perception of the social world as threatening. Parental touch may direct children’s attention away from social threat and increase their trust in others, and thereby help them attend to and explore the broader environment. As such, parental touch may be especially reassuring to those who need it the most: socially anxious children. Social anxiety is among the most common types of anxiety, and typically has its onset in childhood (Bögels et al., 2010). The essential feature of social anxiety is “a marked, or intense, fear or anxiety of social situations in which the individual may be scrutinized by others” (American Psychiatric Association, 2013). Socially anxious individuals fear that others will evaluate them negatively (e.g., depreciate, ridicule, or reject them; Clark and Wells, 1995; Etkin and Wager, 2007; Rapee and Heimberg, 1997). This fear of negative evaluation makes them feel distressed and leads them to avoid social situations. Thus, whereas fear of negative evaluation is the core of social anxiety, social avoidance and distress are consequences of this fear. When socially anxious children are touched by their parent, they may infer that their social environment is less threatening than they thought initially, and thus become less vigilant.

### 1.1 Present study

The present research investigated, for the first time, whether parental touch would reduce children’s social vigilance, especially in socially anxious children. We assessed children’s social anxiety levels, and then randomly assigned parents to touch or not touch their child briefly and gently on the shoulder, right below the deltoid. Parents are naturally inclined to touch the back of the child’s shoulder (Croy et al., 2016; Salt, 1991), and the child allows them to (Suvilehto et al., 2015). We then assessed two pillars of social vigilance: children’s implicit attention to social threat and their trust in unfamiliar others.

We timed our study in late childhood (ages 8–10) and early adolescence (ages 11–14). In late childhood, children spend a lot of time with their parents, feel close to their parents (Larson and Richards 1991), and rely on their parents as a source of safety (Paterson et al., 1995). Upon the transition to adolescence, however, children venture into the world autonomously and seek independence from their parents (Crone and Dahl, 2012; Steinberg and Morris, 2001). From the age of 11, children become less sensitive to parental cues (Gee et al., 2014) and start to side more with their peers than with their parents (Berndt, 1979; Fuligni and Eccles, 1993). Indeed, from this age, children show particularly strong responses to peer disapproval (Moor et al., 2014). Although adolescents may be aware that their parents will be available and responsive in times of need (Allen and Land, 1999), they may be reluctant to rely on their parents as a source of safety. Consistent with this notion, retrospective self-report research found that parental touch in late childhood—unlike parental touch in adolescence—relates to individuals’ later trust in others (Takeuchi et al., 2010). Thus, we hypothesized that the effects of touch would be more pronounced in late childhood than early adolescence.

## 2 Method

### 2.1 Participants

Participants were 138 children ages 8–14 years (*M* = 10.22, *SD* = 1.59; 47% girls, 53% boys; 94% of Dutch origin) and their parent ages 31–59 years (*M* = 43.68, *SD* = 4.84; 52% women, 48% men). Data from 1 child were lost due to technical failure. Participants visited Science Center NEMO, the largest science museum in the Netherlands. The research was part of Science Live, the innovative research program of Science Center NEMO that enables scientists to use NEMO visitors as participants. The study was approved by the ethical committee of the faculty of social sciences of Utrecht University. Parents provided active informed consent, both for their child and for themselves.

There were two experimenters: one was available to the child, another was available to the parent. Children completed all tasks on the computer, in fixed order, and received instructions on their screens. After receiving the touch manipulation, children completed the outcome measures within 10 min.

### 2.2 Social anxiety

While parents were waiting in a separate room, children first completed the 18-item Social Anxiety Scale for Children—Revised (La Greca and Stone et al., 1993), Dutch translation (Koot and Mesman, 2001), consisting of three subscales: Fear of Negative Evaluation (FNE; 8 items, e.g., “I worry that other kids don’t like me”), Social Avoidance and Distress—General (SAD-G; 4 items, e.g., “It’s hard for me to ask other kids to play with me”), and Social Avoidance and Distress—Specific to New Peers or Situations (SAD-New; 6 items, e.g., “I feel shy around kids I don’t know”). The translation has been validated extensively (Reijntjes et al., 2007; Reijntjes et al., 2011) and was readily understood by the children. Responses were given on 5-point scales (1 = *Not at all*, 5 = *All the time*) and averaged across items (FNE: *M* = 2.01, *SD* = 0.74; Cronbach α = .85; SAD-G: *M* = 1.77, *SD* = 0.68; Cronbach α = .61; SAD-New: *M* = 2.30, *SD* = 0.72; Cronbach α = .70). The subscales should be analyzed separately rather than averaged into an overall score (La Greca and Stone et al., 1993). Although we administered all subscales, we focused our primary analyses on fear of negative evaluation, the core of social anxiety. We assessed avoidance and distress to examine the specificity of our findings.

### 2.3 Parental touch

**Fig. 1:**
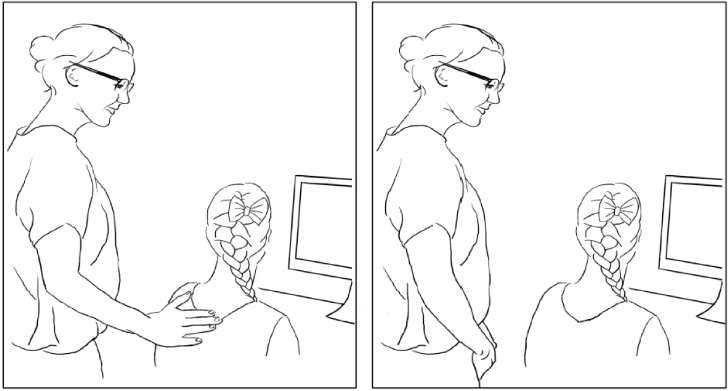
A depiction of the manipulation of parental touch (left panel: touch condition; right panel: no-touch condition). The only difference between conditions was that, in the touch-condition, the parent placed the palm of their right hand gently on the back of the child’s left shoulder, right below the deltoid, for the duration of one second.

Unbeknownst to children, parents were instructed about the touch manipulation in a separate room. The experimenter demonstrated the touch procedure on a child-sized mannequin; parents then practiced the procedure on the same mannequin. When the child had completed the questionnaire, the parent performed the procedure in fixed order: The parent went inside, stood behind the child (on the child’s left side), either did or did not touch the child, told the child that they themselves were going to work on another computer, and walked over to this computer (in the same room, behind a folding screen, out of the child’s sight). Parent-child dyads were randomly assigned to the touch (*n* = 71) or no-touch (*n* = 66) conditions. In the touch condition, the parent placed the palm of their right hand gently on the back of the child’s left shoulder, right below the deltoid, for the duration of one second. In the no-touch condition, the parent performed the same procedure but without touching the child. The manipulation is depicted in Fig. 1.

We designed our manipulation to be precise and subtle, for two reasons. First, its precision ensures that the effects are due to touch itself, not to features that accompany touch (e.g., physical closeness). Second, its subtlety ensures that children do not experience the touch as a deliberate attempt by the parent to console them; indeed, children may not even consciously perceive or remember being touched so subtly.

Experimenters monitored the manipulation. Eleven children were excluded from the analyses because their parents did not execute the manipulation as instructed; the parents (a) provided more physical touch than instructed (e.g., a kiss) or (b) upon entering the room, told the child nothing at all (creating an uneasy silence) or more than was instructed (starting a conversation).

### 2.4 Attention to social threat

When parents were seated behind their own computer, children performed the dot-probe task (Waters et al., 2010). This task indexes how quickly children identify probes that appear at the previous location of social threat (i.e., angry facial expression) or non-threat (i.e., happy facial expression). The more quickly children identify probes that appear at the previous location of social threat, the more their attention is biased toward such threat. Children first performed 10 practice trials.

On each of the 100 actual trials, children were first presented with a fixation point in the center of the screen for 500 ms. Then, two faces appeared for 500 ms, one on the left and one on the right side of the screen. The faces were taken from standardized sets (Ekman and Friesen, 1976; Lundqvist et al., 1998), and were either neutral and angry (40 trials), neutral and happy (40 trials), or both neutral (20 trials, serving as filler trials). An asterisk (i.e., probe) appeared in the location of one of the faces until children responded. On critical trials (i.e., neutral-angry, neutral-happy), the probe appeared in the same location as either the emotional face (congruent trials; 40 trials) or the neutral face (incongruent trials; 40 trials). Children indicated whether the probe appeared on the left or right side of the screen by pressing keys labelled “left” or “right” as quickly and as accurately as possible. Inter-trial interval varied randomly from 750 to 1250 ms.

One child was excluded from the dot-probe analyses because his number of errors (i.e., pressing left when the probe appeared right, or vice versa) exceeded 10% of the trials. The remaining children made an average of 1.18% errors (*SD* = 1.38). Correct responses were analyzed. Reaction times were removed when they were shorter than 200 ms or > 2 *SD*s different from the participant’s own mean (*M* = 3.81% of reaction times removed per participant, *SD* = 1.53). Bias scores were calculated, separately for happy and angry faces, by subtracting the average reaction time on congruent trials from the average reaction time on incongruent trials. Positive values indicate bias to attend to emotional faces, and negative values indicate bias to attend to neutral faces (anger/threat bias: *M* = 6.04, *SD* = 37.17; happiness/non-threat bias; *M* = 2.97; *SD* = 37.97).

### 2.5 Trust

Children were then presented with 30 photos of faces of unfamiliar children (15 boys, 15 girls) with neutral facial expressions, taken from the Child Affective Facial Expression (CAFE) set (LoBue and Thrasher, 2015). Children rated how much they trusted each child (1 = *Not at all*, 4 = *Very much*). Responses were averaged across photos (*M* = 2.49, *SD* = 0.46; Cronbach α = .91).

### 2.6 Touch experience

To index children’s general experience with and enjoyment of parental touch, parents rated how often they cuddle with their child (1 = *Rarely or never*, 5 = *Several times a day*; *M* = 4.22, *SD* = 0.91) and how much the child enjoys cuddling with them (1 = *Not at all*, 5 = *Very much*; *M* = 4.37, *SD* = 0.77). Because parents had to wait until the child had completed the trust ratings, they also completed filler tasks. These tasks were not analyzed because parents were involved as experimenters and were thus aware of the study purpose.

### 2.7 Data analysis

We distinguished between late childhood (ages 8–10, final sample: *n* = 78) and early adolescence (ages 11–14, final sample: *n* = 48) based on a priori cut-offs, established in a similar Dutch sample by an independent research group (Moor et al., 2014). Because our minimal age of inclusion was 8 and NEMO is visited rarely by children over 14 (NEMO Science Museum, 2016), we were unable to select age groups that were farther apart.

Attentional biases were analyzed using Repeated Measures Analysis of Covariance, with bias (threat, non-threat) as within-subjects factor, fear of negative evaluation (continuous, centered) as continuous predictor, condition and age (late childhood, early adolescence) as between-subjects factors, along with their two-, three-, and four-way interactions. Trust ratings were analyzed using hierarchical regression analysis, with condition (0 = no touch, 1 = touch), age (0 = late childhood, 1 = early adolescence), and fear of negative evaluation (continuous, centered) as predictors, along with their two- and three-way interactions. Significance level was set at 0.05. Despite our directional hypotheses, we adhered to two-tailed testing to provide a conservative test at our alpha level.

## 3 Results

Random assignment was successful. Conditions did not differ in children’s sex, age, or social anxiety subscales (fear of negative evaluation, social avoidance and distress—general, social avoidance and distress—specific to new peers or situations), or in parents’ sex or age, *p*s > .356. Table S1 (Supplementary Material) displays correlations between key variables. In the primary analyses, no case unduly influenced the results (all Cook’s distance values < 1).

### 3.1 Attention to social threat

We hypothesized that parental touch would reduce implicit attention to social threat, especially in late childhood. Because fear of negative evaluation formed no main effects or interactions, *p*s > .176, we dropped it from the analyses. There were no main effects or two-way interactions involving bias, condition, or age, *p*s > .264.

**Fig. 2:**
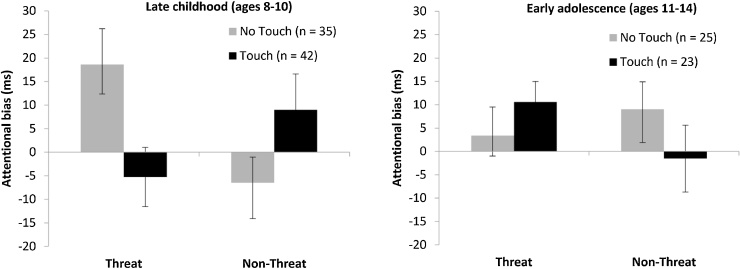
Effects of parental touch on attentional bias for social threat and non-threat in late childhood (left panel) and early adolescence (right panel). Error bars represent standard errors. As noted in the Method section, one child was excluded from the attentional-bias analyses because his number of errors exceeded 10% of the trials.

The critical condition × bias (threat, non-threat) × age interaction was significant, *F*(1, 121) = 8.75, *p = *.004, η_p_^2^ = .07. The condition × bias interaction was not significant in early adolescence, *F*(1, 46) = 2.61, *p = *.113, η_p_^2^ = .05, but was highly significant in late childhood, *F*(1, 75) = 8.36, *p = *.005, η_p_^2^ = .10 (Fig. 2). In late childhood, touch did not affect attentional bias for non-threat, *t*(75) = 1.59, *p = *.115, Cohen’s *d* = 0.37, but significantly decreased attentional bias for threat, *t*(75) = –2.45, *p = *.017, Cohen’s *d* = 0.56. In the no-touch condition, children’s attention was significantly biased toward threat, *t*(34) = 2.46, *p = *.019, Cohen’s *d* = 0.84, but in the touch condition, there was no such bias, *t*(41) = –0.84, *p = *.408, Cohen’s *d* = 0.26. Thus, touch specifically reduced children’s attention to social threat.

### 3.2 Trust

We hypothesized that parental touch would increase trust, especially in late childhood. We examined whether this effect would be especially pronounced for children high in fear of negative evaluation. There were no main effects of condition or fear of negative evaluation, *p*s > .263, but there was a main effect of age, *t*(122) = 2.08, *p* = .040, β = .18, with older children being more trusting. There were no two-way interactions, except a condition × fear of negative evaluation interaction, *t*(119) = 2.25, p = .026, β = .31, with touch weakening the negative association between fear of negative evaluation and trust. However, this effect was qualified by a three-way interaction.

**Fig. 3:**
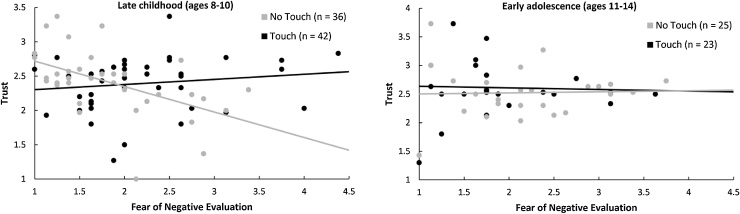
Effects of parental touch on trust in late childhood (left panel) and early adolescence (right panel) depending on children’s pre-existing level of fear of negative evaluation.

The critical condition × fear of negative evaluation × age interaction was significant, *t*(118) = −2.17, *p* = .032, β = −.34. The condition × fear of negative evaluation interaction was not significant in early adolescence, *t*(118) = −0.26, *p* = .795, β = −.06, but was highly significant in late childhood, *t*(118) = 3.14, *p* = .002, β = .55 (Fig. 3). In late childhood, in the no-touch condition, children who feared negative evaluation trusted others less, *t*(118) = −3.27, *p* = .001, β = −.61, but in the touch condition, this association was not significant, *t*(118) = 0.88, *p* = .381, β = .12. Region of significance analysis (Preacher et al., 2006) revealed that, in late childhood, touch *increased* trust in children high in fear of negative evaluation (>0.64 *SD* above the mean) and *decreased* trust in children low in fear of negative evaluation (>0.94 *SD* below the mean).

### 3.3 Auxiliary analyses

#### 3.3.1 Robustness analyses

We examined the robustness of our findings. First, we repeated our main analyses with age as a continuous rather than dichotomous variable (Supplementary Material). One omnibus interaction became marginally significant, but the pattern of results remained the same, and follow-up analyses yielded the same significant effects. Second, we tested whether the findings were qualified by children’s sex, parents’ sex, how often children cuddle with their parent, how much children enjoy cuddling with their parent, and parents’ genetic relatedness to their child (i.e., one grandparent, one adoptive parent, and one stepparent participated). These variables were all unrelated to attentional bias and trust, *p*s > .113, and, when added to our main analyses, did not form four-way interactions with condition, age, and bias (threat, non-threat), or fear of negative evaluation in predicting attentional bias or trust, *p*s > .123.

#### 3.3.2 Specificity analyses

Although we focused our main analyses on the core of social anxiety—fear of negative evaluation—an important question is whether the effects of touch depended on children’s social avoidance and distress; they did not. Social avoidance and distress did not interact with condition, bias (threat, non-threat), or age in predicting attentional bias, *p*s > .203. Social avoidance and distress had no main effects on trust, *p*s > .209, and did not interact with condition or age in predicting trust, *p*s > .296.

#### 3.3.3 Recollection check

After the study, children reported whether they thought their parent had touched their shoulder after entering the room (1 = *Yes*, 2 = *No*, 3 = *I don’t know*). Of children in the touch condition, only 49% accurately reported being touched—not much more than would be expected by chance alone. Importantly, the accuracy of children’s recollections was unrelated to attentional bias and trust, *p*s > .251, and, when added to our main analyses, did not form four-way interactions with condition, age, and bias (threat, non-threat) or fear of negative evaluation in predicting attentional bias or trust, *p*s > .579. Thus, the effects of touch occurred even when children had no conscious recollection of being touched.

## 4 Discussion

We provide, to our knowledge, the first empirical evidence that parental touch reduces children’s social vigilance. We involved parents as experimenters, and we randomly assigned them to touch or not touch their child on the shoulder, right below the deltoid. Parental touch lowered children’s implicit attentional bias for social threat and, among socially anxious children, raised trust in unfamiliar others. These effects occurred only in late childhood, when children still readily rely on their parents for safety.

### 4.1 Theoretical and applied implications

What psychological mechanisms underlie the effects of parental touch? Humans have built-in and largely automatic attentional biases for social threat (Bögels and Mansell, 2004). Over the course of evolution, these biases have served survival by identifying social threat quickly and effortlessly (Öhman and Mineka, 2001). Parental touch may downregulate these social-threat biases by conveying to children that their environment is safe; it may direct children’s attention away from potential threat and toward the broader environment, encouraging them to explore (e.g., Bowlby, 1969/1982Bowlby, 1969/1982). Importantly, parental touch may take different forms depending on whether children face social or physical threat. For example, when children are reluctant to go up to their new classmates (i.e., social threat), parents may touch them on the back of their shoulder to encourage them to approach the threat. By contrast, when children want to cross a busy street (i.e., physical threat), parents may hold them firmly to help them avoid the threat. Compared to social threats, physical threats are typically more urgent and may call for avoidance rather than approach. Supporting the social-physical distinction, socially anxious individuals are hypervigilant for and respond strongly to social but not physical threat (Goldin et al., 2009; Hope et al., 1990).

The psychological consequences of touch are underpinned by a cascade of physiological processes. Being touched by significant others may trigger oxytocin release (Holt-Lunstad et al., 2008; Schneiderman et al., 2012). This rise in oxytocin may activate safety-signaling neural regions, such as the ventromedial prefrontal cortex (Eisenberger et al., 2011), and deactivate threat-processing regions, such as the amygdala and insula (Gordon et al., 2013; Lucas et al., 2015). These effects of oxytocin may be especially pronounced in socially anxious individuals (Gorka et al., 2015; Labuschagne et al., 2010), leading them to reappraise their environment as safe (Stein et al., 2007).

Children may become less sensitive to parental touch upon the transition to adolescence, when they seek independence from their parents and venture into the world autonomously (Crone and Dahl, 2012; Steinberg and Morris, 2001). Consistent with this idea, parental touch reduced children’s social vigilance in childhood but not adolescence. One reason for this developmental change could be that social vigilance is simply less prevalent in adolescence, leaving little room for parental touch to curb it. Our results suggest, for example, that attentional bias for social threat falls and trust rises with age (Fig. S1 and S3).

Parental touch raised trust in socially anxious children, but unexpectedly lowered trust in socially non-anxious children. Children may interpret touch differently depending on their perceptions of the environment. Socially anxious children typically perceive their social environment as threatening (Clark and Wells, 1995; Rapee and Heimberg, 1997). When they are touched by their parent, they may infer that the parent wants to reassure them, conclude that there is little to be afraid of, and trust others more. By contrast, socially non-anxious children typically perceive their social environment as safe. When they are touched by their parent, they may similarly infer that the parent wants to reassure them; yet, because they did not perceive a threat in the first place, they may paradoxically conclude that there is something to be afraid of, and trust others less. Our reasoning concurs with previous findings. First, touch alleviates anxiety in individuals with low self-esteem but not in those with high self-esteem (Koole et al., 2013). Second, overprotective parenting—such as being protective of children, even when they are not anxious—predicts higher social anxiety levels in children over time (Lieb et al., 2000; Wood et al., 2003).

Our findings may inform future intervention efforts. Social anxiety disorder is the most common, yet least often treated, mental disorder in youth (Chavira et al., 2004). In 75% of cases, social anxiety disorder has its onset between ages 8 and 15 (American Psychiatric Association, 2013). Unfortunately, the most commonly used treatment—cognitive-behavioral therapy—is less effective for children with social anxiety disorder than for children with another anxiety disorder (Hudson et al., 2015). Given that parental touch lowers attentional bias for threat and raises trust in socially anxious children, researchers should examine the effects of parental touch on children with social anxiety disorder (e.g., touch given right before children enter novel social contexts, such as their first day at school, a playdate, or a birthday party). Parental touch may help these children overcome their fear and encourage them to expose themselves to novel contexts (Guéguen, 2004). Exposure is one of most effective means to alleviate social anxiety (Feske and Chambless, 1995).

### 4.2 Strengths and limitations

Strengths of our study include its precise experimental manipulation of parental touch, its developmental timing, and its multi-method assessment of children’s social vigilance. Our study also has limitations, which generate novel research directions. First, we examined only the immediate effects of parental touch. Future research should examine how parental touch gets “under the skin” over the course of development. When children are touched sensitively by their parents in novel, anxiety-provoking contexts, they may internalize a sense of safety, and develop the freedom to explore such contexts without the parents’ proximal encouragement (cf. Anisfeld et al., 1990). Second, like previous research (e.g., Feldman et al., 2010), we compared parental touch with no touch at all. Future research should examine how children construe the presence and absence of touch (e.g., whether they perceive the absence of touch as signaling lack of safety).

### 4.3 Conclusion

Our experiment demonstrates, for the first time, that even subtle forms of parental touch can reduce children’s social vigilance.

## Conflict of Interest

None.
