# Supplementary material for: Parental touch reduces social vigilance in children
Source: Dev Cogn Neurosci. 2018 May 9;35:87–93. doi: 10.1016/j.dcn.2018.05.002 (PMC6968960; doi:10.1016/j.dcn.2018.05.002)
Supplement: Supplementary file 1 [file mmc1.docx]

**Supplementary Material**

Table S1 displays zero-order correlations between key variables within each age group and experimental condition. To examine the robustness of our findings, we repeated our main analyses with age as a continuous rather than dichotomous variable. One omnibus interaction became marginally significant, but the pattern of results remained the same, and follow-up analyses yielded the same significant effects. Figures S1, S2, and S3 show how the effects of touch change gradually with age.

**Data Analysis**

Attentional biases were analyzed using multilevel regression analysis, with bias (attention to threat, non-threat) nested within participants, and condition (0 = no touch, 1 = touch) and age (continuous, centered) as predictors, along with their two- and three-way interactions. Trust ratings were analyzed using hierarchical regression analysis, with condition (0 = no touch, 1 = touch), age (continuous, centered), and fear of negative evaluation (continuous, centered) as predictors, along with their two- and three-way interactions.

When there were interactions involving age, we probed them without splitting the sample into separate age groups. That is, we continued to use age as a continuous variable. We did so by conducting simple-effects analyses (Holmbeck, 2002a, 2002b). This method does not split the sample, but rather uses the full sample to estimate effects of touch at different levels along the age continuum. Because our hypotheses pertain to late childhood and early adolescence, we estimated the effects of touch at ages that fall within those age groups (i.e., 2 *SD* below and above the mean of age).

**Attention to Social Threat**

The condition × bias (attention to threat, non-threat) × age interaction was marginally significant, *t*(242) = –1.79, *p* = .074, β = –.24. We conducted follow-up analyses despite its marginal significance, because our hypotheses pertained to specific contrasts, not to the omnibus interaction (Rosenthal & Rosnow, 2009). The condition × bias interaction was not significant in early adolescence (2 *SD*s above the mean of age), *t*(242) = –0.82, *p* = .411, β = –.21, but was significant in late childhood (2 *SD*s below the mean of age), *t*(242) = 2.38, *p* = .018, β = .60 (Figure S1 and S2). Simple effects analysis shows that, in late childhood (2 *SD*s below the mean of age), touch did not affect attentional bias for non-threat, *t*(242) = 1.12, *p* = .265, β = .23, but significantly decreased attentional bias for threat, *t*(242) = –2.25, *p* = .025, β = –.46. Thus, touch specifically reduced children’s attention to social threat.

**Trust**

The condition × fear of negative evaluation × age interaction was significant, *t*(118) = –2.17, *p* = .032, β = –.31. The condition × fear of negative evaluation interaction was not significant in early adolescence (2 *SD*s above the mean of age), *t*(118) = –0.77, *p* = .441, β = –.22, but was highly significant in late childhood (2 *SD*s below the mean of age), *t*(118) = 3.06, *p* = .003, β = .83 (Figure S3). In late childhood, in the no-touch condition, children who feared negative evaluation were more distrusting, *t*(118) = –2.83, *p* = .006, β = –.82, but in the touch condition, this association was not significant, *t*(118) = 0.67, *p* = .507, β = .08. Region of significance analysis (Bauer & Curran, 2005; Preacher, Curran, & Bauer, 2006) revealed that, in late childhood, touch *increased* trust in children high in fear of negative evaluation (> 1.11 *SD* above the mean) and *decreased* trust in children low in fear of negative evaluation (> 0.78 *SD* below the mean).

**References**

Bauer, D. J., & Curran, P. J. (2005). Probing interactions in fixed and multilevel regression: Inferential and graphical techniques. *Multivariate Behavioral Research, 40*, 373-400. doi:10.1207/s15327906mbr4003_5

Holmbeck, G. N. (2002a). Post-hoc probing of significant moderational and mediational effects in studies of pediatric populations. *Journal of Pediatric Psychology, 27*, 87-96. doi:10.1093/jpepsy/27.1.87

Holmbeck, G. N. (2002b). *Addendum: Post-hoc probing of significant moderational and mediational effects in studies of pediatric populations.* Unpublished manuscript, Loyola University.

Preacher, K. J., Curran, P. J., & Bauer, D. J. (2006). Computational tools for probing interaction effects in multiple linear regression, multilevel modeling, and latent curve analysis. *Journal of Educational and Behavioral Statistics, 31*, 437-448. doi:10.3102/10769986031004437

Rosenthal, R., & Rosnow, R. L. (2009). *Contrast analysis: Focused comparisons in the analysis of variance.* New York, NY: Cambridge University Press.

Table S1

*Zero-Order Correlations Between Key Variables Within Each Age Group and Experimental Condition*

|  | 1 | 2 | 3 | 4 | 5 | 6 |
| --- | --- | --- | --- | --- | --- | --- |
| Late childhood (*n* = 36 and 42 in no-touch and touch conditions, respectively) | | | | | | |
| 1. FNE | — | .21 | .36* | -.07 | .04 | -.52** |
| 2. SAD-G | .36* | — | .44** | .09 | -.03 | .05 |
| 3. SAD-New | .38* | .46** | — | -.23 | -.07 | -.25 |
| 4. Threat bias^a^ | -.12 | .28 | -.15 | — | .03 | .37* |
| 5. Non-threat bias^a^ | -.11 | -.10 | -.19 | .02 | — | -.09 |
| 6. Trust | .15 | .16 | .05 | -.02 | -.13 | — |
| Early adolescence (*n* = 25 and 23 in no-touch and touch conditions, respectively) | | | | | | |
| 1. FNE | — | .31 | .51** | -.35 | -.01 | .03 |
| 2. SAD-G | .67*** | — | .46* | .16 | .12 | .11 |
| 3. SAD-New | .50* | .73*** | — | .06 | .09 | -.08 |
| 4. Threat bias^a^ | -.13 | .07 | .15 | — | .16 | -.28 |
| 5. Non-threat bias^a^ | .29 | .27 | .13 | .17 | — | -.31 |
| 6. Trust | -.04 | -.18 | -.18 | -.12 | -.28 | — |

*Note.* FNE = Fear of Negative Evaluation. SAD-G = Social Avoidance and Distress—General. SAD-New = Social Avoidance and Distress-Specific to New Peers or Situations. Correlations within the no-touch condition are displayed above the diagonal; correlations within the touch condition are displayed below the diagonal.

^a^As noted in the Method section, one child was excluded from the attentional-bias analyses because his number of errors exceeded 10% of the trials.

**p* < .05. ***p* < .01. ****p* < .001.

*
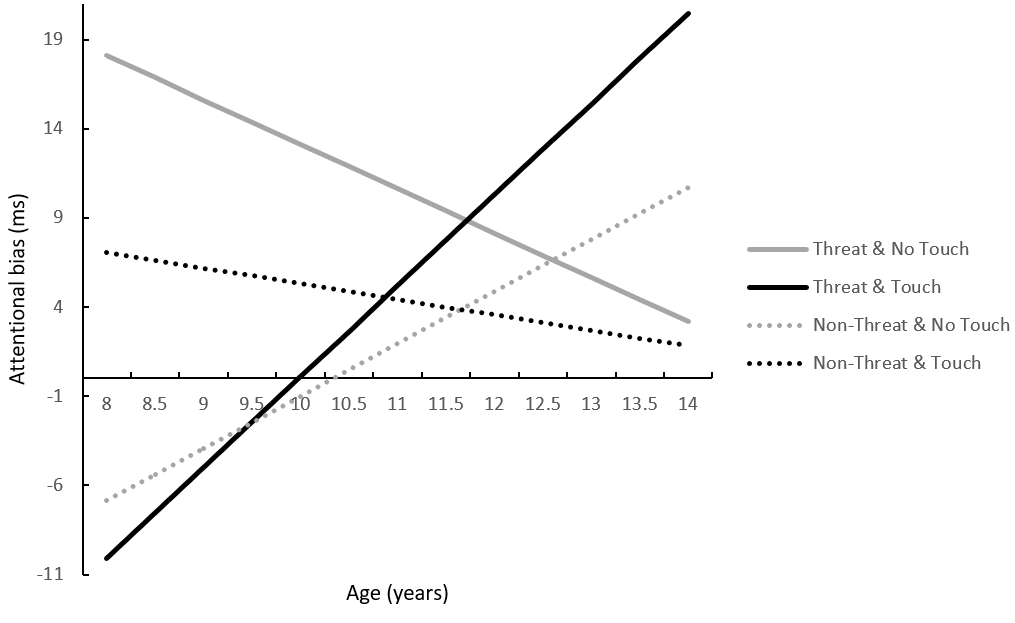
*

*Figure S1.* Line graph displaying the effects of parental touch on attentional bias for social threat and non-threat across age. We estimated the effects at different levels of age using the full final sample (*N* = 125, with 60 and 65 children in the no-touch and touch conditions, respectively). As noted in the Method section, one child was excluded from the attentional-bias analyses because his number of errors exceeded 10% of the trials.


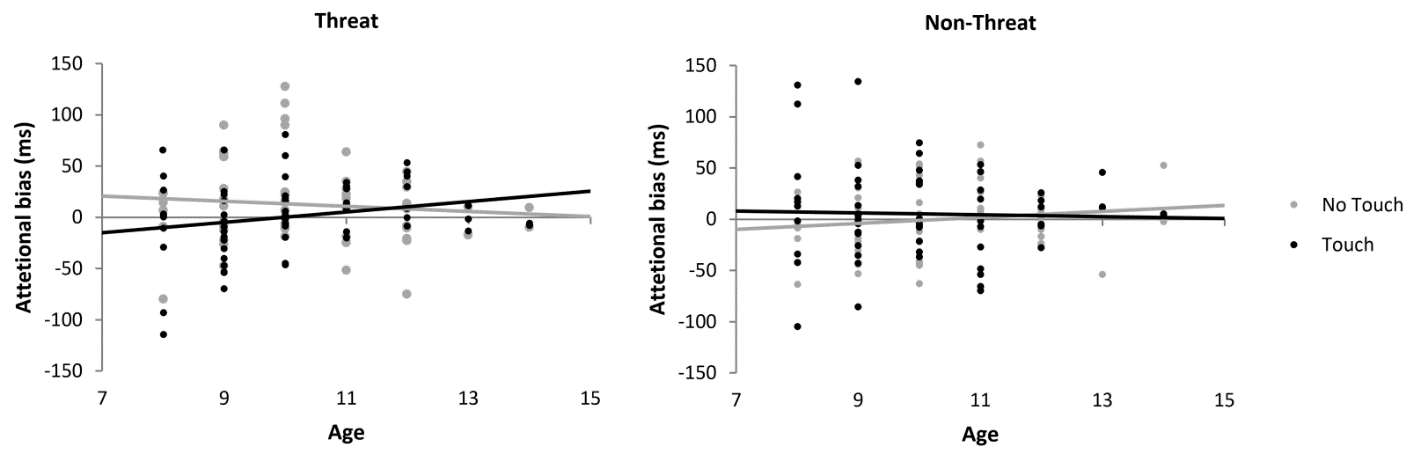


*Figure S2.* Scatterplot displaying the effects of parental touch on attentional bias for social threat and non-threat across age. We estimated the effects at different levels of age using the full final sample (*N* = 125, with 60 and 65 children in the no-touch and touch conditions, respectively). As noted in the Method section, one child was excluded from the attentional-bias analyses because his number of errors exceeded 10% of the trials.

*
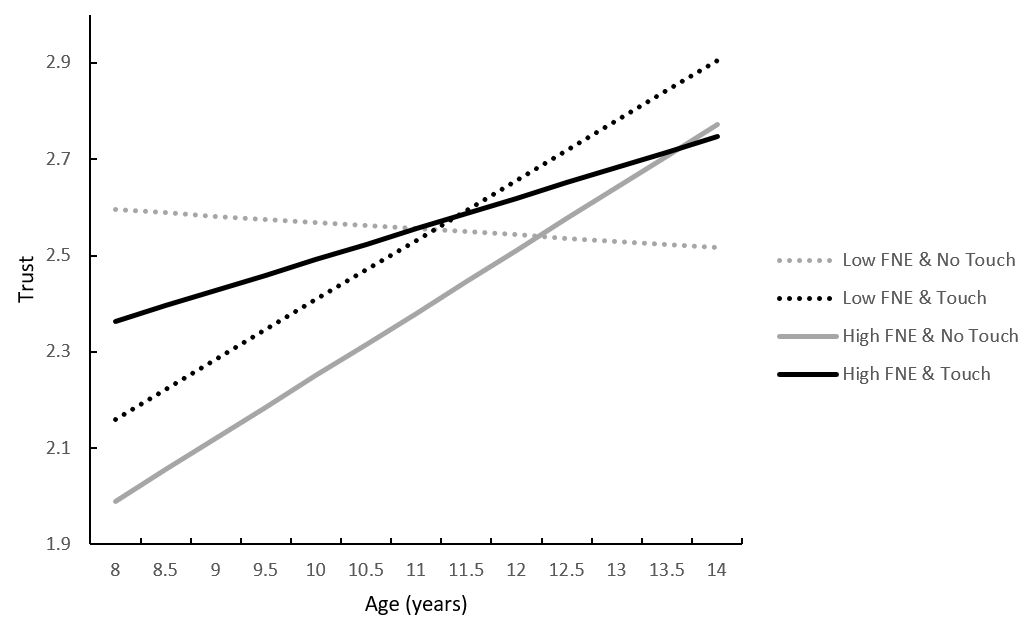
*

*Figure S3.* Effects of parental touch on trust across age depending on children’s pre-existing level of Fear of Negative Evaluation (FNE). We estimated the effects for those with high FNE (1 *SD* above the mean) and low FNE (1 *SD* below the mean) at different levels of age using the full final sample (*N* = 126, with 61 and 65 children in the no-touch and touch conditions, respectively).
